# The ‘triple win’ practice: the importance of organizational support for volunteer endeavors undertaken by health care professionals and staff

**DOI:** 10.7189/jogh.08.010305

**Published:** 2018-06

**Authors:** Dilani Logan, Lawrence C Loh, Vivian Huang

**Affiliations:** 1 Duke University, Durham, North Carolina, USA; 2 Dalla Lana School of Public Health, University of Toronto, Toronto, Canada; 3 Médecins Sans Frontières, New York, New York, USA

Volunteering abroad has become a popular activity within the global health space among health care trainees and providers. When performed well, volunteering can create positive community change by filling service gaps and providing access to technical expertise that would be otherwise unavailable. In turn, volunteer participants benefit by using and developing core skills and knowledge; networking and building professional contacts; and attaining personal satisfaction with a feeling of improved sense of self-worth. For employers, as well, volunteering work has been shown to forestall employee burnout and increase employee engagement.

Despite these myriad benefits, participation in such endeavors by providers faces several barriers, ranging from a lack of financial support, the need to maintain credentials, inadequate work-life balance, and policies that prohibit extended leaves of absence. This paper demonstrates that the adoption of a Corporate Social Responsibility (CSR) model could help employers, participants, and communities abroad reap the benefits of a ‘triple win’ ideology within the health care sector.

The ‘triple win’ ideology believes that these three stakeholder groups can benefit when health care professionals are empowered to take extended leaves of absence for humanitarian work. Healthcare professionals improve their personal skills and gain experience in other settings, and can experience less burnout; employers have more satisfied and revitalized employees and an improved public reputation for global community service; and most importantly, host communities benefit from the mitigation of harms associated with poorly considered shorter-term work and can receive quality services and/or capacity and infrastructure building and development provided by experienced professionals that can address identified community challenges and priorities.

In unpacking and examining the ‘triple win’ ideology, improved institutional policies that enable health care providers and trainees to participate in longer-term volunteer missions will pay dividends for all involved, while carefully recognizing that such experiences must be done well and responsibly in order to truly derive the proposed benefits.

Community involvement through volunteering is a strong societal value in North America. From neighborhood clean-ups in elementary school to the mandated completion of volunteer hours in high school and service learning courses in college, the importance of giving back and being an involved citizen is repeatedly emphasized throughout our formative years.

Volunteering can create positive community change by filling service gaps, helping the disenfranchised, and providing access to expertise that would otherwise not be possible. Volunteers also benefit from an increased understanding of community needs and issues; the utilization and development of core skills and knowledge; the creation of new friendships and professional contacts; and the attainment of personal satisfaction supporting a sense of self-worth [1].

For these reasons, one might imagine that volunteering beyond one’s formative years would be of interest to communities and individuals. However, research shows entry into the workforce results in lower participation in volunteer activities, entangled amongst the numerous responsibilities and commitments of working life. In many cases, the irony is that individuals are now “experts” with specific knowledge and skillsets that could be better applied to address community and global issues, but lack the time or resources to do so.

Volunteering in global health and development typically includes engagements that involve expertise from medical volunteers and those involved in public health and related fields. Efforts range from clinical assistance to providing education and mentorship or providing tools/equipment to foster community growth and development [2]. Motivations can vary, but broadly speaking, volunteers “act of their own free will, with a greater goal in mind, be it moral objective, a sense of personal achievement, or even a strategic career plan” [3]. Among health care professionals, in particular, interest in volunteering abroad on “medical missions” or “global health experiences” has grown over the past decade. However, participation in such volunteer efforts faces several impediments, which can be summarized as financial, professional credentialing, work-life, and organizational barriers. Many health care professionals carry hefty loans after training which restrict their available time and resources for volunteering, and must maintain their credentials through dedication to continuing medical education credits, re-certification exams, and professional development training sessions among other things. Furthermore, as volunteering periods are considered to be separate from one’s professional career or development, volunteer efforts are generally not seen to be contributors to one’s career or their affiliated employer. Work-life balance and organizational expectations also pose challenges. Because organizational expectations and demands have greatly increased juxtaposed to shrinking budgets, lengthy patient census and increased service requirements, medical professionals are often discouraged from volunteer activities.

However, it should be noted that these issues are directly relevant to the North American context. Applied to short-term volunteering stints abroad that last less than three months, it is important to note that there are variations in policy amongst different countries. For instance, during the Ebola crisis in 2014, the Dutch government sent out a call to chief hospital administrators across the Netherlands to inform and facilitate the voluntary deployment of medical providers to affected countries, this included full benefits such as health insurance, logistics and salary as if working in the Netherlands [4]. The government funded the secondments of medical professionals and 50 specialized laboratory technicians in response to Médecins sans Frontières’ call for personnel at West African treatment centers [4]. While this example was started as in response to an emergency situation, the outcomes of the program ultimately turned into a longer-term response that spanned two years. Given its success, the program could be replicated for non-emergency contexts.

**Figure:**
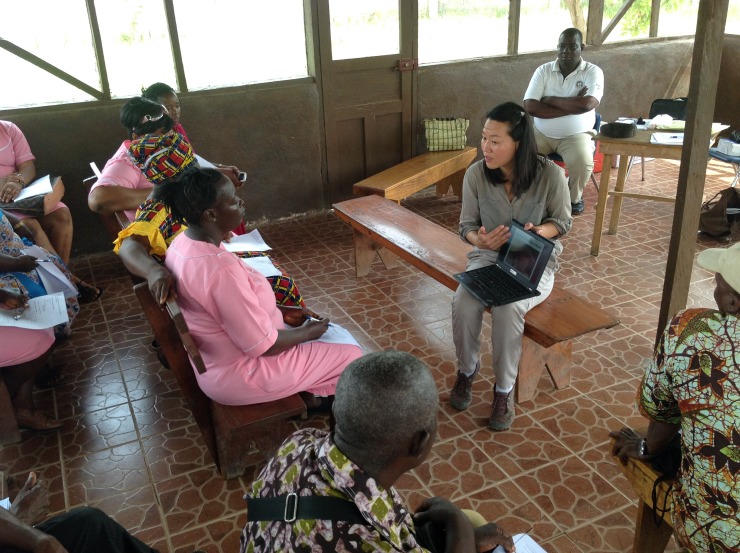
Photo: from the collection of Dr Vivian Huang (used with permission)

One might argue that the desire among medical professionals to volunteer is there, but these barriers prevent their active participation and dedication over the longer-term. Particularly among those health care professionals who wish to volunteer in global health and development efforts, these barriers have driven many to pursue short-term “medical missions” abroad during their limited vacation and free time instead. Such ventures have faced increasing criticisms as being unethical and unsustainable, and researchers are increasingly attempting to characterize the potential contributions, drawbacks and possible harms of such efforts to communities abroad. Short term volunteering endeavors create a culture of instability amongst the community as the local economy shifts to make way for the ever-changing flux of volunteers, which inadvertently takes away from the local economy in times of crises and prevents opportunities for culturally sensitive, longer-term mutually beneficial exchange between local professionals, communities and expatriate volunteers. Related to this is the genuine need for volunteer health care workers to support other, more compatible humanitarian endeavors as well as long-term capacities.

Healthcare in North America traditionally has not been enthusiastic in supporting experienced staff to undertake longer-term humanitarian roles. Many practitioners struggle to gain employer support necessary to pursue volunteering efforts abroad. Often, taking up a longer-term volunteering stint comes at the expense of loss of pay, job security, and reputational risk as extended leaves to volunteer can risk hard-earned career security at home.

That said, the knowledge that health professionals possess could vitally support the work of development agencies and organizations to assisting communities abroad to enhance primary health care, drive immunization campaigns, improve disease surveillance, optimize clinic and hospital-based care, enhance rehabilitation and feeding programs, etc.

The emergence of Corporate Social Responsibility (CSR) as a concept has encouraged many businesses in other industries and professionals to incorporate volunteering into their efforts to contribute positively towards the betterment of society. Research from other industries has found that CSR goes beyond encouraging economic growth alone and uses existing social structures to meet modern-day challenges, such as substantially controlling and mitigating the effects of HIV/AIDS, creating reductions in poverty and building human capital [5].

While health care has lagged in incorporating CSR, certain employers in the space increasingly subscribe to the concept of the ‘triple win’ ideology, which believes that the active participation of health care practitioners, North American employers, and in-need communities could be wholly beneficial for all parties involved. Each party presents particular needs that could be addressed through the participation of the remaining parties, and by carefully integrating and involving the participation of all three parties, a ‘triple win’ is achieved whereby: health care professionals increase personal experience and possibly shielded from career burnout; vulnerable communities are empowered to strive for healthier lives as a result of improved medical access and knowledge, support and quality of care from experienced professionals; and North American employers have a more satisfied workforce with improved public reputation.

Interested practitioners who are supported in volunteering are able to benefit and grasp opportunities through service expansion, self-actualization, and decreased risk of career burnout. Specific to global health and development efforts, humanitarian volunteering efforts enable clinicians to collaboratively exchange knowledge and practices with new colleagues in the host community and extend their skillset in areas where it is most needed. There are additional benefits of self-fulfillment with improved and sharpened intercultural competency/sensitivity and analytical skillsets.

The second stakeholder benefiting from the ‘triple win’ are the host communities. Recipients of carefully considered volunteer efforts can facilitate community access to timely expert care, professional development and collaboration opportunities/exchanges with local practitioners. In-need communities also enjoy an opportunity to demonstrate and share their customs, traditions and practices with visiting volunteers. Cultural exchanges of customs, traditions and practices that occur in the field, can be used by volunteers to enhance their practices at home when interacting with multicultural populations including colleagues and patients, further extending this benefit to multiple groups.

Arguably, one of the most important benefits are the hidden and unrealized “wins” for the employers themselves. Adopting corporate social responsibility (CSR) proposals by publicly traded US companies has been found to lead to significant financial performance through increases in profitability ratios [6]. It could be posited that these increases result from factors that could be applied to the health care sector such as improved employee performance and increased patient engagement. Supporting employees to pursue humanitarian and sustainable global health efforts provides a plethora of benefits for health care institutions. By preventing employee burnout, institutions can realize gains in physician and employee retention including saving money in training new providers (it is estimated that for each physician that burns out and leaves their job, it can cost the institution up to US$ 500 000 dollars) and alleviates the burden on individual practitioners to make perceived ultimatum decisions between volunteering their skillset abroad or staying domestically focused [7]. Longer-term, international opportunities also work to increase cultural sensitivity and humility, which can provide improved patient interactions at home. Working in international settings with communication barriers pushes volunteers to augment their communication skills with similar patient populations at home by improving their ability to utilize simplistic language and rely on non-verbal communication skills such as eye-contact and hand-touching [2]. Both are important aspects of patient-provider interactions and has the potential to result in improved care.

Certainly, there are challenges to ensuring interested employees are supported in their undertaking of volunteering or humanitarian efforts, and there are possible harmful effects with longer-term volunteering opportunities as well. However, given the benefits described, one might imagine that such support, while more challenging, will likely pay dividends in the long run. Traditional management approaches that discourage or fail to support health care practitioners in volunteering ignores the potential ‘triple win’ that could be achieved by implementing employer-supported, employee volunteer programs within the health field.

To that end, health care administrators/leaders can start to consider fostering an organizational culture that is supportive of volunteer and humanitarian endeavors abroad for their practitioners over the course of their careers. Such a paradigm could engage staff, institutions, and countries and communities in need [8]. With all-around “wins” for all parties involved, it is incumbent for us to return to that societal value of volunteering and determine how the health care community can best encourage the growth of thoughtful volunteering and humanitarian assistance efforts to support global health and development priorities.
